# EHMTI-0013. The relations between attention deficit and hyperactivity disorder and different types of headaches in a non- clinical sample of adolescents

**DOI:** 10.1186/1129-2377-15-S1-B10

**Published:** 2014-09-18

**Authors:** J Genizi, D Marom, I Srugo, N Kerem

**Affiliations:** 1 Pediatric Neurology Unit, Bnai Zion Hospital, Haifa, Israel; 2 Pediatric Department, Bnai Zion Hospital, Haifa, Israel; 3 Adolescent Medicine Unit, Bnai Zion Hospital, Haifa, Israel

## Introduction

Stress is considered to be a major trigger for aggravation of headaches. In a previous study we demonstrated a high prevalence of ADHD among patients who were referred to a pediatric clinic due to headaches. In the present study we examined whether this is true for the general population of adolescents.

## Aims

To assess the prevalence of primary headaches among school students and the relation to learning disorders and ADHD.

## Methods

A Computerized questionnaire that was filled out anonymously by tenth grade students attending a high school in Haifa, after receiving informed consent from parents and informed ascent from the students participating in the study.

## Results

Out of 310 valid questionnaires, 230 students (81%) complained about headaches (88% of the girls and 76% of the boys, p=0.08), 98 of them (43%) elaborated on the characteristics of their headaches: 50% matched migraine, 28% Tension Type Headache, and in 22% there was not enough data to make a definitive diagnosis. Out of the students who had headaches, 27% were diagnosed with ADHD and 32% with learning disabilities. Students who felt as if they had ADHD and or learning disabilities but were not diagnosed formally with these diagnoses had significantly more headaches than their diagnosed peers (p=0.002).

## Conclusions

Our work indicates that students who feel that they have learning disabilities and or ADHD but were not diagnosed, complain more about headaches compared to their peers who were either diagnosed or did not feel they had one of the two diagnoses.

No conflict of interest.

